# Bitpacking techniques for indexing genomes: II. Enhanced suffix arrays

**DOI:** 10.1186/s13015-016-0068-6

**Published:** 2016-04-23

**Authors:** Thomas D. Wu

**Affiliations:** Department of Bioinformatics and Computational Biology, Genentech, Inc., 1 DNA Way, South San Francisco, CA 94080 USA

**Keywords:** Enhanced suffix array, Sequence alignment, Genomics, Data compression

## Abstract

**Background:**

Suffix arrays and their variants are used widely for representing genomes in search applications. Enhanced suffix arrays (ESAs) provide fast search speed, but require large auxiliary data structures for storing longest common prefix and child interval information. We explore techniques for compressing ESAs to accelerate genomic search and reduce memory requirements.

**Results:**

We evaluate various bitpacking techniques that store integers in fewer than 32 bits each, as well as bytecoding methods that reserve a single byte per integer whenever possible. Our results on the fly, chicken, and human genomes show that bytecoding with an exception guide array is the fastest method for retrieving auxiliary information. Genomic searching can be further accelerated using a data structure called a discriminating character array, which reduces memory accesses to the suffix array and the genome string. Finally, integrating storage of the auxiliary and discriminating character arrays further speeds up genomic search.

**Conclusions:**

The combination of exception guide arrays, a discriminating character array, and integrated data storage provide a 2- to 3-fold increase in speed for genomic searching compared with using bytecoding alone, and is 20 % faster and 40 % more space-efficient than an uncompressed ESA.

**Electronic supplementary material:**

The online version of this article (doi:10.1186/s13015-016-0068-6) contains supplementary material, which is available to authorized users.

## Background

High-throughput sequencing [1] makes it critical to accelerate the alignment of query reads to a genome, and influences the design of genomic data structures for fast pattern search. Although pattern search is germane to many fields, genomic search is of such fundamental and timely importance in biology that accelerating genomic search is worthwhile in its own right. Furthermore, genomics is a distinctive domain, characterized by (1) very large texts, such as the 3 billion base pairs in the human genome; (2) a small alphabet size, with only four nucleotides and a possible fifth character to represent ambiguity or uncertainty; (3) various types of repetition in the genomic text, on both small and large scales; and (4) the need to handle mismatches and gaps between the query read and the genome, which can require searches on short substrings in a query and result in large numbers of matches in a genome.

In a companion paper, we show how hash tables for representing genomes can be made faster by introducing novel bitpacking compression techniques, which allow for larger *k*-mers and therefore higher specificity. In this paper, we consider the prevailing alternative to hash tables, namely, suffix arrays [2] and related variants. A suffix array (SA) represents a genome sequence as a list of positions, arranged according to the lexicographic ordering of their corresponding suffixes. Suffix arrays are used in such programs as segemehl [3], last [4], mummer [5], reputer [6], star [7], and as an initial stage in recent versions of gsnap [8], which also employs hash tables for more complex alignments.

For suffix arrays, as with any representation, there is a tradeoff between time and space. Uncompressed representations generally offer the fastest retrieval times, but require the most space, and therefore may not fit within the available amount of memory on a given computer. Accordingly, many proposals for compressing suffix arrays have been advanced [9]. We are interested in investigating how such methods work in genomics applications. For the purpose of genomic search, we do not necessarily need all of the functionality of a suffix array, but can consider representations that support only the task of pattern matching.

In general, pattern matching can be considered as two related tasks: (1) counting the number of pattern matches, and (2) locating their positions in the target text. For most applications in genomics, the locating task is the relevant one, which yields all genomic positions that match a given query read or substring in the read. Table 1 demonstrates how suffix arrays perform on both tasks, in the two columns labeled SA. Two possible suffix array algorithms for pattern matching are reverse search, which uses a successor array called Psi, and forward search, which performs a binary search through the suffix array entries. More detailed descriptions of these and other established algorithms can be found elsewhere [13].

**Table 1:** Speed of genomic search and space usage

		Reverse search				Forward search				
		SA	CSA-Sada	SA-WT	CSA-WT	SA	ESA	ESA-bp	ESA-byte	ESA-gdi
Space used (bytes per genome length)										
Fly		3.5	0.9	4.9	0.9	3.5	11.4	8.0	6.6	7.1
Chick		3.8	1.0	5.1	0.9	3.8	11.9	8.5	6.3	6.8
Human		4.0	1.0	5.5	0.9	4.0	12.4	9.0	7.1	7.6
Counting task (microseconds per query)										
Fly	12-mers	42.9	45.6	1.6	1.7	46.8	2.3	52.6	5.2	1.7
	24-mers	81.5	88.8	4.7	4.8	47.6	2.5	54.0	5.3	2.1
	36-mers	119.1	130.0	5.9	7.0	42.5	2.8	54.6	5.6	2.2
Chick	12-mers	52.4	44.5	2.0	2.1	54.7	2.8	61.4	7.3	2.3
	24-mers	98.0	90.3	4.5	5.3	53.5	3.6	65.7	8.7	3.0
	36-mers	148.1	137.4	9.0	8.2	54.5	3.8	67.1	7.9	3.3
Human	12-mers	59.8	49.3	2.4	2.7	58.4	3.4	75.0	10.0	3.1
	24-mers	101.5	94.5	5.8	5.6	66.8	5.1	76.7	12.1	4.0
	36-mers	151.0	139.3	9.3	9.4	70.2	5.2	81.1	11.9	4.0
Locating task (microseconds per query)										
Fly	12-mers	49.6	223.8	21.5	147.8	52.0	3.5	52.2	6.7	2.9
	24-mers	86.5	135.0	12.0	45.2	47.4	3.1	53.9	5.5	2.5
	36-mers	130.2	157.6	12.9	32.1	51.3	3.1	59.3	6.5	2.4
Chick	12-mers	62.0	682.7	45.8	628.3	61.5	5.2	63.9	10.7	4.3
	24-mers	104.5	102.9	7.2	16.3	54.2	4.0	64.1	7.9	2.8
	36-mers	141.0	134.5	8.8	13.5	51.7	3.8	65.6	7.7	3.3
Human	12-mers	80.1	6300.8	602.6	5798.1	99.0	30.4	94.1	37.6	28.5
	24-mers	101.4	393.6	40.8	264.0	68.1	6.8	77.4	13.8	5.5
	36-mers	149.7	175.8	12.6	46.1	60.0	5.1	79.3	12.7	4.3
Locating task (nanoseconds per match result)										
Fly	12-mers	69.2	312.4	30.1	206.2	72.5	4.8	72.9	9.3	4.1
	24-mers	360.5	562.8	50.2	188.6	197.8	13.0	224.6	23.1	10.3
	36-mers	941.9	1140.1	93.8	232.7	371.5	22.8	429.3	47.0	17.3
Chick	12-mers	36.7	404.1	27.1	371.9	36.4	3.1	37.8	6.3	2.5
	24-mers	2993.0	2948.3	206.9	467.4	1551.5	115.8	1836.9	226.7	81.5
	36-mers	9723.0	9274.2	606.4	928.8	3566.7	259.6	4527.3	533.2	226.3
Human	12-mers	3.3	259.9	24.9	239.1	4.1	1.3	3.9	1.6	1.2
	24-mers	71.0	275.6	28.6	184.8	47.7	4.7	54.2	9.7	3.9
	36-mers	800.8	940.7	67.7	246.5	320.8	27.3	424.5	68.2	23.2

Genome sources: Drosophila melanogaster version 5.25.64 (Fly), Gallus gallus gg4 (Chick), and Homo sapiens hg19 (Human)

* SA* uncompressed suffix array; *CSA-Sada* compressed suffix array using Sadakane method; *SA-WT and CSA-WT* uncompressed and compressed suffix array, respectively, using wavelet tree. *ESA* enhanced suffix array, *ESA-bp* ESA with balanced parenthesis representation; *ESA-byte* ESA with bytecoding; *ESA-gdi* ESA with exception guide arrays, discriminating character array, and integrated data structure

A standard suffix array can be improved by increasing search speed, reducing memory requirements, or both. To reduce memory usage, techniques have been developed for compressing the array, such as the proposal by Sadakane [10] or wavelet trees [11], which contain bitmaps for subsets of the genomic alphabet. These methods are benchmarked as CSA-Sada and CSA-WT in Table 1, as well as using the wavelet tree component with an uncompressed suffix array, shown as method SA-WT. The methods above are available in a widely used library called the Succinct Data Structure Library (SDSL) 2.0 [12].

The scope of this paper does not permit a full description of these various compression methods. However, the data clearly show that both space and retrieval time can vary widely, depending on which representation method is used. Therefore, for our goal of accelerating genomic search speed, it is critical to choose an appropriate genomic representation, as allowed within the amount of available memory.

If sufficient memory is available, we can augment the suffix array with auxiliary data structures to create an *enhanced suffix array* (ESA) [14]. The ESA auxiliary data structures are the longest common prefix (LCP) array and the child array. The LCP array records the length of the common substring between adjacent entries in the suffix array, and allows a search algorithm to search against the common region when comparing a query sequence against the suffix array. Common prefixes impose a hierarchical, or parent–child, structure upon the suffix array. For each set of suffix array entries that share a common prefix, the character following the prefix serves to partition the set into smaller children subsets. The child array records where each subset begins, and allows a search algorithm to traverse the hierarchy of common prefixes efficiently.

The drawback of an ESA is the extra space required for the LCP and child arrays. These arrays are similar in size to the suffix array, so they offer a worthwhile opportunity for space savings. For example, a straightforward representation of the suffix array, LCP array, and child array requires 12 GB each for the human genome, with its 3 billion base pairs. Although modern computers often have large amounts of primary random-access memory, or RAM, compression of data structures can still have benefits. In particular, smaller data structures can improve the effectiveness of caching, in which recently used blocks of data are kept temporarily in ultrafast storage for subsequent use.

Accordingly, compression of the LCP and child arrays has been investigated by others. For example, one proposal for compressing the LCP array permutes the order of elements in text order, rather than in suffix array order [15], which allows the permuted array to be represented by monotonically increasing values. Another proposal represents the child array as a bitmap representation of balanced parentheses [16], which is benchmarked in Table 1 as ESA-bp.

As an alternative to these relatively complex compression schemes, we consider a simpler technique called bitpacking, in which integers are represented with fewer than the 32 bits that they are normally allocated. The idea of bitpacking itself is not new, and bitpacking techniques, such as Elias delta codes and Fibonacci codes, have been applied to suffix arrays in the literature [17]. However, vectorized bitpacking, which uses parallel arithmetic operations available on modern computers, represents a recent area of research, and is shown in our companion paper to be particularly useful for offset arrays in genomic hash tables. It is therefore worth considering whether such bitpacking methods could be applied to LCP or child arrays.

A technique similar to bitpacking is bytecoding, either using variable numbers of bytes for each integer, or using a fixed number of bytes, with exceptionally large values stored in a separate array. In fact, a bytecoding scheme for the LCP and child arrays was proposed in the original paper on enhanced suffix arrays [14]. We can consider this to be a reference benchmark, and we show the results of this approach for genomes in Table 1 as ESA-byte, which is relatively fast for both counting and locating tasks.

The goal of this paper, then, is to explore whether improvements upon ESA-byte are possible for genomic search. Our end result is shown in Table 1 as the rightmost column, method ESA-gdi. The acronym “gdi” stands for three concepts introduced in this paper, namely, an exception guide (EG) array for accelerating bytecoding; a discriminating character (DC) array for streamlining the ESA forward search algorithm; and an integrated data structure appropriate for storing related LCP, DC, and child array values. These enhancements result in a genomic search method that reduces space usage for the LCP and child arrays, is faster than the ESA-byte benchmark, and is both faster and more space-efficient than the native uncompressed ESA.

## Methods

### Suffix arrays

A suffix array is an indexed representation of a genome [2]. Although we use the term “genome”, our methods can be used generally to represent any target sequence or set of sequences, such as a set of genomic contigs or transcripts, that serve as a reference for alignment. In this paper, we will use a running example based on the genomic string $$S = acaaacatat{\$}$$, an example introduced by others [14]. A genomic string *S* of length *n* consists of nucleotides drawn from the alphabet $$\Sigma _S = \{a, c, g, t, x\}$$, and ends with a special terminating character “$”. We use the character “x” to represent a nucleotide whose identity is unknown or considered irrelevant for search, such as in the repetitive end regions of chromosomes called telomeres.

**Fig. 1:**
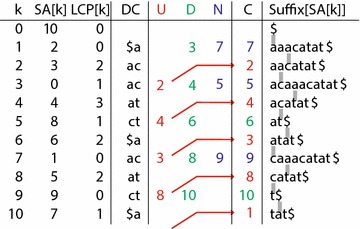
Enhanced suffix array tables for the string *acaaacatat*$. Values are shown for the suffix array *SA*, longest common prefix *LCP* array, and the Child *C* array, consisting of Up *U*, Down *D*, and Next *N* values, with Next values taking precedence over Down values. Up values can be stored in the preceding array position. These values are* colored* to correspond with the* arrows* in Fig. 2. The suffixes in lexicographic order are given by $$\text{Suffix}[\text{SA}[k]]$$. The discriminating character (DC) array introduced in this paper represents the first characters that differ between two adjacent suffixes at $$\text{Suffix}[\text{SA}[k-1]]$$ and $$\text{Suffix}[\text{SA}[k]]$$. The locations of these characters among the suffixes are shown as* grey bars*

In our convention, positions in a string are indexed starting with 0, so the terminating character “${\$}$” is located at position *n*. A genomic string has $(n+1)$ suffixes, $\text{Suffix}[0]$ through $\text{Suffix}[n]$, where $\text{Suffix}[k]$ represents the substring *S*[*k*..*n*]. These suffixes can be sorted lexicographically, giving rise to a suffix array $\text{SA}[k]$, which represents the starting positions of the suffixes after the sort. In our exposition, the terminating character “${\$}$” is considered to have the lowest lexicographic order, preceding “a”. The sorted suffixes and corresponding suffix array for our example are given in Fig.  1, in the columns labeled Suffix and SA.

A suffix array can be used by itself to search for a given query string *P* of length *m*. The query string consists of nucleotides drawn from the alphabet $\Sigma _P = \{a, c, g, t\}$, which excludes the character “x”, since uncertain readings are generally rejected by sequencing machines, so all of *P* is typically known unambiguously. (In the rare event of an uncertain character reported in the query string, hash table methods could be used for pattern matching.) The search process involves a binary search through the suffix array to find indices *i* and *j*, such that $\text{Suffix}[\text{SA}[i]]$ and $\text{Suffix}[\text{SA}[j]]$ are the narrowest pair of suffixes that enclose *P* lexicographically. If *i* and *j* exist and $i\le j$, the values of $\text{SA}[i]$ through $\text{SA}[j]$ give the positions in the genome that match *P*. In other words, the values $\text{SA}[i]$ through $\text{SA}[j]$ represent the solution for the locating task in pattern search, while the quantity $(j-i+1)$ represents the solution for the counting task. A binary search process can find all occurrences of a query string *P* in a genome in $O(m\log n)$ time.

### Compression of bucket arrays

The binary search process can be boosted by using a bucket array [2], which stores the starting and ending indices *i* and *j* in the suffix array for each possible *q*-mer drawn from $\Sigma _P$, for some well-chosen value *q*. Then, if the query string *P* has sufficient length $m\ge q$, a constant-time lookup of the *q*-mer in the bucket array can replace the first *q* steps of the binary search. Because the bucket array is a series of successive pointers into the suffix array, it is amenable to differential coding techniques, such as those that we explored for offset arrays in our companion paper. However, if *q* is sufficiently small, typically with a value of 12 or less, where a single array of indices for 12-mers occupies only 67 MB, compression may not be so critical. (Since $\Sigma _S$ also contains the character “x”, which is not included in any *q*-mer, we must explicitly store two arrays of indices, one for the starting index *i* and one for the ending index *j,* to encode the lcp-interval [*i*..*j*] for each *q*-mer, entailing a total storage space of 134 MB.) Since bucket arrays are well established as a technique for accelerating pattern search, we will not evaluate their effectiveness in this paper, or even employ them. Instead, we will investigate other techniques for accelerating genomic search and reducing memory usage. In the sections below, we describe various techniques to be evaluated computationally in this paper.

### Compression of the LCP array

Search using a suffix array involves comparing characters in the pattern against entries in the suffix array. This comparison process can be made more efficient by using an LCP (longest common prefix) array [2]. The LCP value at index *k* is the longest prefix shared between the suffix at $\text{SA}[k]$ and the one at $\text{SA}[k-1]$, shown in Fig. 1 as the column labeled LCP. The LCP array can speed up search in practice by allowing one to skip some of the character comparisons needed to differentiate among candidate suffixes when they share a common prefix. Instead, we can compare the pattern characters directly against the common prefix, as represented by any one of the candidate suffixes, to determine whether the prefix matches. Whereas a suffix array by itself can find all occurrences of a pattern *P* in $O(m\log n)$ time, with the addition of an LCP array, search can be performed in $O(m+\log n)$ time.

An LCP array generally contains small values, and these values can be bitpacked in various ways, including a direct coding scheme as used in vectorized bitpacking. In such a scheme, we encode arrays of integers in blocks of 64, each with a uniform bit width as needed to represent the largest integer in the block. Various versions of block-based bitpacking were developed and evaluated in our companion paper for representing offset arrays in genomic hash tables. However, that application required differential coding, or encoding differences between elements of a vector of ascending values. In contrast, although LCP values are not monotonically increasing and hence not amenable to differential coding, they are nevertheless candidates for direct coding.

In direct coding, we could conceivably arrange the integers of each block in any layout, including the BP64-horizontal, BP64-vertical, or BP64-columnar layout, as described in our companion paper. Since extracting a single value does not require parallel loads or additions, SIMD operations are unnecessary. Rather, a separate scalar procedure is needed for each possible bit width (i.e., the 16 even bit widths used in BP64), and for each possible entry $x_i$ within the block (64 entries in BP64). Hence, it would seem that implementation in each of the three layouts would require $16\times 64 = 1024$ separate access procedures. However, if we use the BP64-vertical layout and consider the contents of the bitpacked array to be a series of 32-bit words, rather than 128-bit vectors, the 64 entries can be reduced to 16 distinct cases, where $x_i$ selects a procedure based on $\lfloor x_i/4\rfloor$, applied to the series starting at one of the four starting words in the block, as determined by the quantity $(x_i \bmod 4).$ This scheme reduces the number of procedures to be $16\times 16 = 256$.

Because values in an LCP array are generally small, another option is to use a bytecoding scheme, as proposed by others [14]. In this scheme, each LCP value can be stored in a single byte, if it is less than 255. If the LCP value is 255 or greater, then a value of 255 is used as a flag to indicate that the actual value is stored in a separate data structure for exceptions. Although exceptions could be represented in a true hash table, where the key *k* is evaluated using a hash function to determine the corresponding bucket of possible key/value pairs, a simpler representation stores key/value pairs in a single array, in ascending order of their keys. Such an array of exceptions can then be processed by binary search.

Alternatively, by adding an *exception guide (EG) array*, we can restrict the scope of the binary search to a portion of the exception array, effectively making the search process closer to that of a true hash table. An EG array contains the corresponding location in the exception array at regular intervals of *k*. For example, for a guide interval of 64, the array would store the position of the first exception that satisfies $k\ge 64$, $k\ge 128$, $k\ge 192$, and so on. Therefore, given a key *k*, we would divide the key value by the guide interval, look up that entry in the EG array, and then obtain the approximate subsection to search in the exception array. We would then require only a small sequential or binary search through that subsection. (Our implementation uses binary search.) An exception array plus an EG array achieves the same behavior as a true hash table, with the hash function being division by the guide interval. In fact, an EG array is analogous to a bucket array, which stores pointers to starting positions in a suffix array, at intervals corresponding to successive *q*-mers. However, as far as we can tell, guide arrays have not been proposed in the literature for bytecoding representations, and are not currently implemented within SDSL 2.0, perhaps because exceptions are typically not as prevalent as they are in genomics applications.

Although bitpacking or bytecoding of small LCP values already provides a compact representation, these values can be compressed even further by using wavelet trees [11], which use bit arrays as a representation. Another general approach to representing integer arrays is called directly addressable variable-length coding (DAC) [18], which allows for a variable number of bytes.

Another possible technique for compressing an LCP array is to permute it [15]. A permuted LCP array, or PLCP array, contains the LCP values in text order *a*, rather than in lexicographic order *k*, satisfying $\text{PLCP}[a] = \text{PLCP}[\text{SA}[k]] = \text{LCP}[k]$. One way to represent this PLCP array is by a succinct bitarray [15] that can be queried using a select function, which is how this method is implemented within SDSL. Alternatively, it has been observed [10] that the sequence of values $\text{PLCP}[a] + a$ is nondecreasing, making it amenable to differential coding techniques such as those used in vectorized bitpacking. In that compression scheme, to retrieve $\text{LCP}[k]$, we would look up $a = \text{SA}[k]$, perform differential decoding of the value located at position *a*, and subtract *a* from the result. Applicable differential coding methods include the BP64-vertical, BP64-columnar, or BP32-columnar formats discussed in our companion paper, as well as existing universal code formats.

### Compression of the child array

The speed of pattern matching can be improved further by adding a child array, which represents the hierarchical structure of common prefixes in a suffix array (Fig. 1). A child array allows search to be done in $O(m|\Sigma _S|)$ time, independent of the text length, and dependent instead on the length *m* of the pattern and the size of the alphabet. Therefore, for genomic search, where the text length can be quite large, the child array can be particularly effective. Also, for genomic applications, the alphabet size is small, limited to the four nucleotides and a character for an unknown or ambiguous nucleotide. A formal exposition of the child array can be found elsewhere [14]; for our purposes, we aim to provide some intuition for the data structure and its possible improvements.

**Fig. 2:**
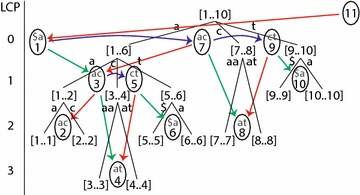
LCP-interval tree for the string *acaaacatat*$. The lcp-intervals are shown in *square brackets*, with *lines* pointing to their children intervals. Intervals are separated by $$\ell$$-indices, shown as integers at the bottoms of *ovals*. Pairs of letters are shown above each $$\ell$$-index, indicating its discriminating characters. Both lcp-intervals and their $$\ell$$-indices are arranged vertically by their LCP-value, and horizontally by their index. Child relationships among $$\ell$$-indices are shown as* arrows* to the* left* (in* red*) for Up;* right* (*green*) for Down; and curved arcs (*blue*) for Next values, to correspond with the* colors* in Fig.  1

A suffix array can be searched as a hierarchy, consisting of sets of entries with common prefixes. Each set, or lcp-interval [*i*..*j*] represents a range in the suffix array from index *i* through *j*, having a common prefix of length $\text{lcp}_{ij}$. The search process begins with the entire suffix array [1..*n*] and progressively narrows the range of indices, based on matches from the query string. The set of lcp-intervals can be conceptualized as a tree, although this tree is virtual and not constructed explicitly (Fig.  2). In this figure, each lcp-interval is shown as a bracketed range of numbers. A parent lcp-interval is connected to child lcp-intervals with branches, each of which is labeled with a substring. The leaves of the tree consist of singleton lcp-intervals where $i=j$, representing individual entries in the suffix array.

In pattern search, each lcp-interval represents a decision branch point, with a child interval to be selected based on a character from the query string *P*. Selection is based on the branch labels in the lcp-interval tree, as shown in Fig.  2, which is a virtual representation and not stored explicitly. The first parent interval is [1..*n*], and in our example, its child interval is selected based on the first letter of *P*, or *P*[0]. The child interval [1..6] is selected if *P*[0] is “a”; [7..8] if it is “c”, and [9..10] if it is “t”.

At a given parent lcp-interval [*i*..*j*], the search process selects from among the child lcp-intervals

$$\begin{aligned}{}[i..(k_1-1)], [k_1..(k_2-1)], \ldots , [k_t..j] \end{aligned}$$

or perhaps none of them, in which case the pattern match fails. The child lcp-intervals are separated by the $\ell$-indices $k_1$, $k_2$, ..., $k_t$, which, combined with *i* and *j*, are sufficient to define the child lcp-intervals for a given parent. Hence, $\ell$-indices are essentially decision breakpoints in the suffix array at various levels of the pattern comparison process. The $\ell$-indices are shown in Fig.  2 as the bottom number inside each oval.

A critical observation is that the children $\ell$-indices for a given parent lcp-interval are at the same level, and accordingly have a common LCP value $\text{lcp}_{ij}$ for the prefix shared among all suffixes from $\text{SA}[i]$ through $\text{SA}[j]$. In other words,

$$\begin{aligned} \text{LCP}[k_1] = \text{LCP}[k_2] = \cdots = \text{LCP}[k_t] \end{aligned}$$

Therefore, the relevant character from the query string *P* needed to select a child lcp-interval is the character located at $\text{lcp}_{ij}$, or $P[\text{lcp}_{ij}]$. In our example in Fig.  2, the parent lcp-interval [7..8] has a single child $\ell$-index of 8, having an LCP value of 2. Selection between the child lcp-intervals [7..7] and [8..8] depends on the character *P*[2] in the query string. If *P*[2] is “a”, the child lcp-interval is [7..7]; if it is “t”, the child lcp-interval is [8..8]; and for all other cases, pattern matching fails.

Because each $\ell$-index sits between two child lcp-intervals, each of which is selected by a particular character, we can associate those characters with each $\ell$-index. We propose to call these the *discriminating characters* for a given $\ell$-index. In our example, the $\ell$-index of 8 can be labeled with the discriminating characters “a” and “t”, as indicated by the letters “at” in the top of the oval for $\ell$-index 8. The discriminating characters for a given $\ell$-index *k* are the first characters that differ between the suffixes $\text{Suffix}[\text{SA}[k-1]]$ and $\text{Suffix}[\text{SA}[k]]$, as shown by the gray bars in the suffixes of Fig.  1. Since these two suffixes share a common prefix of length $\text{LCP}[k]$, the discriminating characters are located in the genomic string at $S[\text{SA}[k-1] + \text{LCP}[k]]$ and $S[\text{SA}[k] + \text{LCP}[k]]$. The discriminating characters can be organized in a DC array in order of the $\ell$-indices, as shown in the column marked DC in Fig.  1. These discriminating characters represent a novel contribution of this paper, which we shall use later to accelerate the ESA search algorithm.

The $\ell$-indices have relationships among themselves. The relationship from one child $\ell$-index to its sibling can be represented as a Next value, shown as curved blue arrows in the Fig.  2. For example, the Next sibling for index 1 is index 7, and the Next sibling for index 7 is index 9. The first child $\ell$-index $k_1$ for a parent interval [*i*..*j*] is given either as a Down value (green) from index *i* or an Up value (red) from index $(j+1)$. The directions Down and Up indicate whether the child $\ell$-index is larger or smaller, respectively, than the starting index. In the figure, since the $\ell$-indices are arranged horizontally by ascending value, a Down relationship is depicted by a green arrow to the right, and an Up relationship by a red arrow to the left. To avoid treating the initial lcp-interval [1..*n*] as a special case, we consider the $\ell$-index $(n+1)$ to have an Up relationship to the $\ell$-index 1.

To compress a child array, we note from [14] that all relevant Next, Down, and Up values can be stored in a single array (Fig.  1), by allowing Next values to take precedence over Down values, and storing an Up value at index $k_{a}$ at slot $(k_{a}-1)$ in the array. Furthermore, as further proposed by [14], the values in a child array can be reduced by representing them as differences relative to their original index, as $\text{Next}[k] - k$, $\text{Down}[k] - k$, or $k - \text{Up}[k]$. Also, because index values differ by at least one, we can subtract an additional 1 from each of these differences. One proposal for compressing this array has been to use a bytecoding scheme, where the values are stored as a vector of bytes, with values of 255 or greater stored in an exception array, to which we can add an EG array. However, as with the LCP array, small integer values can also be directly coded using a vectorized bitpacking scheme. Alternatively, a more complex approach to compression is a balanced parenthesis representation [16, 19], which can then be represented compactly as a bitmap, where an open parenthesis is encoded by 1 and a close parenthesis by 0. This bitmap can then be navigated with auxiliary data structures that facilitate performing rank and select operations and finding matching open and close parentheses.

### Branch lookup

So far, we have discussed various methods for compressing the LCP and child arrays, which we will evaluate empirically. Our second line of analysis considers ways to streamline the ESA search algorithm, as presented in Algorithm 1, which is adapted from Algorithm 6.8 in [14].

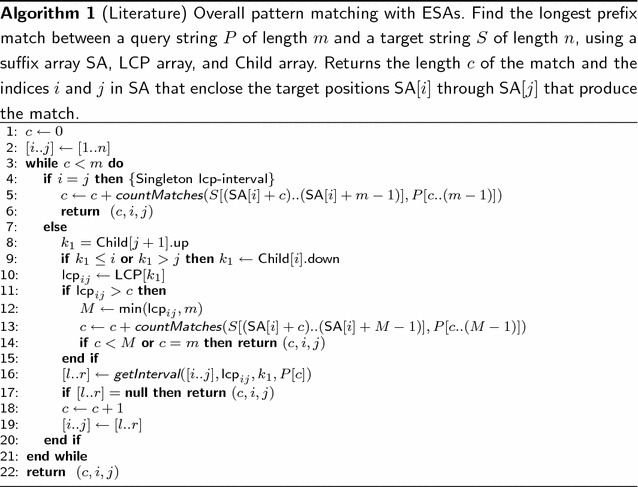



In this algorithm, ESA pattern search is based on repeated steps of finding the child lcp-interval that matches the appropriate character $P[\text{lcp}_{ij}]$ from the given query string *P* against the alternatives at each successive parent lcp-interval. At the end of the search, the algorithm returns the length *c* of the longest prefix match between *P* and substrings in *S*, and the indices *i* through *j* in the suffix array that enclose those matches. If *c* equals *m*, then the entire query string matches; otherwise, only part of the query string does. The algorithm relies upon a procedure *countMatches* that counts the maximum number of matches in some suffix of *P* by comparing it to lookups from the target string *S*. The auxiliary LCP and child arrays in the ESA are designed to minimize the need to count character matches explicitly until the end of the search process, when a final set of positions cannot be discriminated any further. Instead, most of the search process consists of traversing the child array, using a procedure *getInterval*, which finds the child lcp-interval corresponding to a given character $p = P[c]$ from the query string. The algorithm for *getInterval* is presented in Algorithm 2, based on Algorithm 6.7 in [14].

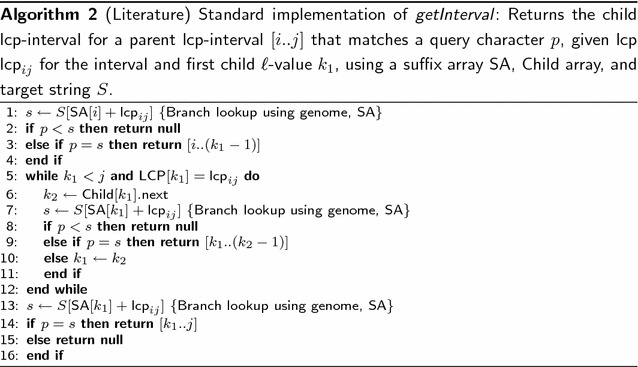



In order to make pattern search more efficient, we focus on lines 1, 7, and 13 of Algorithm 2, which retrieve a character from each of the child suffixes for testing against the desired query character *p*. Since these characters can be found in the branch labels of the lcp-interval tree, we call this process *branch lookup*. The standard, or genome-based, implementation of branch lookup consults the genomic string *S* to obtain the various alternatives. However, this lookup process can be costly for genomics applications, where jumps in suffix array entries over a long genomic text make it unlikely that any given character is stored in cache memory from a recent lookup. Furthermore, genomic strings are often bitpacked, either using 2 bits per character or 3 bits in order to represent the ambiguous character “x” (as in our implementation of gmap [20]), so accessing the genomic string can involve time-consuming shifting and masking operations. Finally, finding the desired position in the genomic string requires a lookup in the suffix array $\text{SA}$, which can be costly because its large size also makes caching unlikely, and if the suffix array is compressed, lookup consumes time to decompress each suffix array element.

One way to reduce the number of lookups in the genome and suffix array is to use the discriminating character (DC) array we introduced previously. A revised algorithm for *getInterval* that uses a DC array is shown in Algorithm 3. In this DC-based approach, a given $\ell$-index *k* yields a corresponding DC array value $\text{DC}[k] = (s_1,s_2)$ containing two branch characters $s_1$ and $s_2$ that can be used to select the child lcp-interval (line 1). In our example, recall that the $\ell$-index of 8 has a DC value of “at”. We can use the fact that $s_1$ and $s_2$ are in lexicographic order to facilitate the initial comparison between the query and the genome (lines 2–5), as follows: (1) if $s_1 < p < s_2$, then *p* was skipped and *getInterval* can return null, indicating that no match was found, and likewise (2) if $p < s_1$ for the first child lcp-interval or $p\ne s_2$ for the last child lcp-interval, *getInterval* can return null. Subsequent accesses to the DC array (line 11) are useful only for obtaining the value of $s_2$. But in all cases, each access to the DC array replaces potentially expensive accesses to both the suffix array and the genome text.

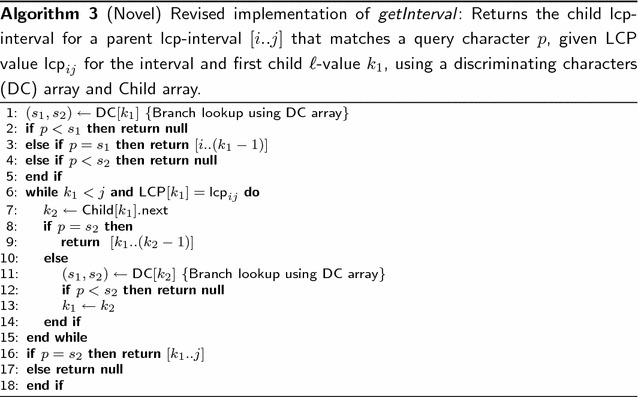



For genomes, we can store the entries of a DC array efficiently; in fact, each possible DC pair can be stored in 4 bits, or a nibble. Recall that the alphabet $$\Sigma _S$$ for the genomic string consists of the nucleotides a, c, g, t, and x. Because the two characters in a DC pair must be in lexicographic order, there are 15 possibilities for a DC pair in the genomic domain: $a, $c, $g, $t, $x, ac, ag, at, ax, cg, ct, cx, gt, gx, and tx. A nibble is sufficient to represent all 15 possibilities. We can pack two nibbles into a byte, so for a genomic string of length *n*, the DC array occupies *n* / 2 bytes.

### Integrated data structure

Our third concept for increasing ESA-based genomic search speed involves improving memory access. Because of memory caching in CPUs, in which recently retrieved memory and surrounding data are kept temporarily in ultrafast storage, procedures can be made faster by placing related data next to one another, when it is anticipated that they will be needed at approximately the same time. In pattern search, the child value for $k_1$ and its LCP value are needed sequentially (lines 8–10 in Algorithm 1 and lines 6–7 in Algorithm 3). Likewise, the DC values are also needed at this time (lines 1 and 11 in Algorithm 3).

Therefore, it makes sense to integrate the LCP, child, and DC data structures, so that $\text{LCP}[k]$, $\text{Child}[k]$, and $\text{DC}[k]$ are retrieved in the same cache line. We have discussed previously that the LCP and child arrays can be represented in a bytecoding format with exceptions and an optional EG array. It is therefore relatively straightforward to interleave the bytes for $\text{LCP}[k]$ and $\text{Child}[k]$ into a single combined array. As for the DC array, a single byte in the DC array represents the two nibbles for two adjacent $\ell$-indices. Therefore, we can include the DC array as well using an integrated data structure that stores auxiliary suffix array information in blocks of 5 bytes: two adjacent LCP values, two adjacent child values, and one byte representing two adjacent DC pairs. The exception array and EG array for the bytecoded LCP and child arrays are stored in separate data structures.

The idea of interleaving data structures is not new. Proposals have been made to combine the suffix array and LCP array in raw or compressed format [21, 22]. However, that format does not account for the child array used in ESAs. With our introduction of the DC array, accesses to the suffix array are eliminated during iterations of selecting child intervals, so interleaving of the suffix array is no longer beneficial in our scheme. Rather than incorporating the suffix array, our integrated data structure includes only the LCP, child, and DC arrays that are closely linked in Algorithms 1 and 3.

## Evaluation

### Experimental setup

We evaluated the speed and space usage of various compression and algorithmic methods by computational experiments. We used genomes of different sizes, namely, the fly genome (D. melanogaster version 5.25.64), chicken genome (Gallus gallus version 4), and human genome (version hg19). Although dedicated software is available for constructing enhanced suffix arrays, such as *mkvtree* (http://www.vmatch.de) and *mkESA* [23], we wanted to augment the ESA approach and to compare ESA methods against other techniques for genomic search. Therefore, we based our experiments and implementation on the Succinct Data Structure Library (SDSL) 2.0 package, which is publicly available as C++ source code [12], but which we have augmented with code for our methods and experimental benchmarks. The augmented version is available for download as Additional file 1, which is the same source code as provided in our companion paper on bitpacking for hash tables. Genomic input files for our benchmarking experiments are hosted on a public Web site, with downloading instructions available within the package. Alternatively, we have prepared a package, available for download as Additional file 2, that allows users to generate their own benchmarks from any DNA or RNA source.

Our additional methods for suffix arrays are implemented in five classes: bp64_vlc_vector, which provides direct coding of integer arrays with a block size of 64; byte_guide, which implements bytecoding of integer arrays, with or without an EG array at a user-specified interval; genome_esa, which builds an enhanced suffix array representation of a genome; genome_discrim, which builds a discriminating character (DC) array for a given genomic suffix array; and genome_integrated, which provides a data structure that interleaves the DC, LCP, and child arrays, with the LCP and child arrays represented using bytecoding with an EG array.

Our benchmarking programs evaluate these classes and native SDSL methods in retrieving LCP values, selecting a child at an lcp-interval, and performing genomic search. Details of each benchmarking program are provided in individual sections later. However, in general, for each benchmarking program, data structures were either read into memory from the filesystem or generated de novo in memory from the input files. A checksum was computed over the results to ensure that the methods gave consistent results and that the compiler did not optimize out the query.

All timing experiments were performed on a reserved Linux computer having 32 Intel Xeon E5-2667 v3 8-core processors running at 3.20 GHz. The computer had total memory of 264 GB and cache memory of 20 MB. The SDSL 2.0 library was compiled with the GNU g++ compiler, version 4.9.0, with the default settings, which turned off debugging code, and added the compiler flags “-O3 -ffast-math -funroll-loops -msse4.2”. Unless specified otherwise, all experiments were repeated for 9 trials, with each trial generating different random values and testing different compression strategies in a randomly selected order. Results are summarized by the median over the trials. We also measured the time for iterating through each dataset, obtaining the query and performing the checksum, and subtracted the median times from all runs. These times amounted to a negligible fraction of the overall running times.

### Retrieval of LCP values

**Fig. 3:**
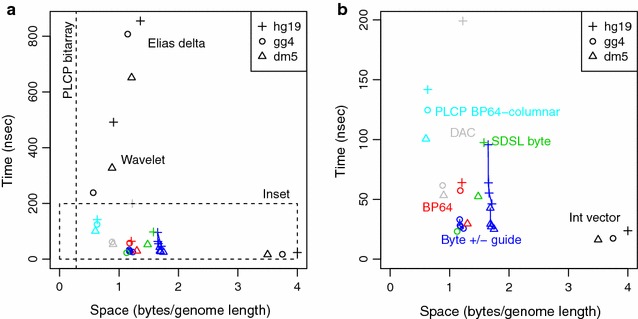
Timing results for LCP retrieval. Genomes tested are fly (dm5), chicken (gg4), and human (hg19). *a* Overall time and space usage. **b** Zoomed image of fastest methods, as bounded by the *horizontal dashed line* in graph (**a**). Graphs plot the time in nanoseconds per query as a function of the space required in bytes per genome length. Formats tested are: integer vectors (Int vector); permuted LCP (PLCP) array using a succinct bitarray; direct coding using Elias delta encoding; wavelet trees; differential coding of the PLCP using a BP64-columnar format; direct coding in blocks of 64 (BP64); bytecoding as implemented within SDSL (SDSL byte); and our implementation of bytecoding with or without an exception guide array at an interval of 1024, 256, or 64. Data points for the bytecoding format are joined by lines for each genome, where bytecoding without an exception guide has the slowest time within each group, and a guide interval of 64 has the fastest time. The *vertical line* in (**a**) indicates the space usage of permuted LCP, since its running time lies outside the bounds of the graph

For retrieval of LCP information, the task in pattern search is to find $\text{LCP}[k]$, for a given entry *k* in the suffix array. We therefore implemented a benchmarking program that retrieves the LCP value for 1 million random indices drawn uniformly in the range of 1 through the genome length *n*. We tested various methods for storing the LCP array, as described in the Methods section, with the overall results shown in Fig.  3a, and an enlarged plot of the inset shown in Fig.  3b.

The most basic storage format is the int_vector class in SDSL, which corresponds closely to an uncompressed array, except that a uniform bit width can be used over the entire array. The SDSL encoding uses 28, 30, and 32 bits, respectively, for the fly, chicken, and human LCP arrays. Retrieval times for int_vector arrays are 16–24 ns/query, but they occupy approximately 3.5–4 bytes per array element. (An uncompressed array using 32 bits for each array element is not shown, but gives approximately the same retrieval times.)

At the other end of the spectrum, an implementation of a permuted LCP array using the lcp_support_sada class in SDSL requires the smallest amount of space, at 0.28 bytes/entry, or 7 % of the space for an uncompressed array. However, retrieval times are extremely slow, at 8963 ns/query, 11,173 ns/query, and 276,050 ns/query for the fly, chicken, and human genomes, respectively. These large values for time do not fit within our graph for Fig.  3a, so we represent only their space usage with a vertical dashed line. Figure 3a also shows space and time measurements for the Elias delta and wavelet tree methods for LCP arrays, implemented as lcp_vlc and lcp_wt, respectively. The Elias delta encoding provides compression of 30–35 % compared with the uncompressed array, but retrieval times that are 36–47 times slower. The wavelet tree method is both smaller and faster, with space usage at 15–25 % and retrieval times that are 14–21 times slower than the uncompressed array.

Faster ways for retrieving compressed LCP values are shown in more detail in Fig.  3b. We implemented a bitpacking method for representing a permuted LCP array using our BP64-columnar method, as implemented in the class bp64_encc_vector. We obtain space usage of 16–17 % and retrieval times that are 6–7 times slower than an uncompressed array. We applied the directly addressable variable coding (DAC) method from SDSL class lcp_dac, and obtain relatively fast retrieval for the fly and chicken genomes (3.3–3.6 times slower than the uncompressed array), but slow retrieval times for the human genome (8.4 times slower). A direct encoding of LCP values using the BP64 bitpacking method yields compression of 30–37 % and retrieval times that are 1.8, 3.3, and 2.7 times slower than the uncompressed array for the fly, chicken, and human genomes, respectively.

We also evaluated the bytecoding scheme in which LCP values less than 255 are stored in a vector of bytes, and values of 255 and greater are stored in an exception array, with or without an EG array. We find that the rate of exceptions differ across the three genomes, with the fly genome having 8.6 % and the human genome having 8.1 % of their LCP values being 255 or greater, but the chicken genome having only 2.2 % of its values being exceptional. This different characteristic of the chicken genome is reflected in the graph values for bytecoding, where the chicken genome requires about 0.5 bytes/genome length less storage than the fly or human genomes. As we discuss later, the chicken genome is remarkable for having relatively little noncoding DNA or duplicate DNA.

We benchmarked both our own implementation of bytecoding without an EG array and the SDSL implementation called lcp_byte. The SDSL implementation is slightly faster for the fly genome and slightly slower for the chicken genome than our implementation. Within our own implementation, bytecoding by itself is slightly faster than BP64 for the chicken genome, but slower for the fly and human genomes. This can be explained by the greater percentage of exception values in fly and human, which causes more time to be spent in binary search through the array of exceptions.

However, when an EG array is added, time for binary search is reduced, and bytecoding provides the fastest retrieval times of any compression method. For an EG array with an interval of 1024, the retrieval times are 1.6–2.7 times that of the uncompressed array; for a guide interval of 256, the times are 1.6–2.3 times slower; and for a guide interval of 64, the times are 1.5–2.0 times slower. A guide interval of 64 requires slightly more space than larger guide intervals, with the total compression being 33–50 % of the uncompressed array, compared with 31–48 % for bytecoding alone. Since the guide adds negligible space, there appear to be few drawbacks to using EG arrays with bytecoded data.

### Selection of child lcp-intervals

Selection of a child lcp-interval corresponds to lines 8–10 and 16 in Algorithm 1, with various data structures tested for these lines and the *getInterval* procedure. Benchmarking for this task involved 1 million random parent lcp-intervals, each drawn from a set of pre-compiled genomic 12-mers, plus a corresponding random query character to select a possible child lcp-interval. We excluded all singleton lcp-intervals, which have no child lcp-intervals. For SDSL methods, we used the child selection procedure from the library when available. We computed the time to return either the matching child lcp-interval or an indication that no such match exists. For all child formats, the LCP array was represented using the lcp_bitcompressed format from SDSL.

**Fig. 4:**
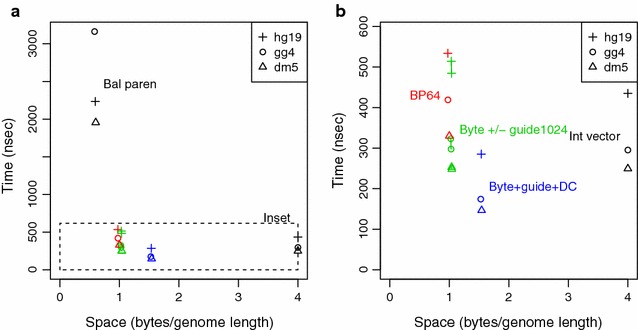
Timing results for child interval selection. Genomes tested are fly (dm5), chicken (gg4), and human (hg19). **a** Overall time and space usage. **b** Zoomed image of fastest methods, as bounded by the *horizontal dashed line* in graph (**a**). Formats tested are: storage of the relative child values as integer vectors (Int vector); balanced parentheses (Bal paren); direct coding in blocks of 64 (BP64); bytecoding with and without an exception guide array at an interval of 1024; and bytecoding with an EG array and a discriminating character (DC) array. Data points for the bytecoding format are joined by lines for each genome, where bytecoding without a guide has the slower time within each group

The child array can be stored as an uncompressed SDSL int_vector, occupying 4*n* bytes, or approximately the same as the uncompressed suffix array and LCP array. The uncompressed child array gave selection times of 249 ns/query for fly, 295 for chicken, and 435 for human (Fig.  4). We applied the balanced parenthesis method [16, 19] by using the cst_sada template with the csa_bitcompressed and lcp_bitcompressed classes to avoid compression of the suffix or LCP array, and therefore isolate the effect of the balanced parenthesis representation. We obtained a compact space representation that was 15 % of the uncompressed child array. However, selection times were slow at 5–11 times that of the uncompressed array (Fig.  4a).

The inset is shown in more detail in Fig.  4b. We applied bitpacking using the BP64 scheme and obtained selection times that were 1.2–1.4 times those of the uncompressed child array. Bytecoding of the child array resulted in selection times that were 1.0–1.2 times those of the uncompressed array. Adding an EG array with an interval of 1024 resulted in a 2–9 % speedup. Smaller guide intervals (not shown) had only a minimal improvement in speed beyond that. The smaller effect of the EG array for child values, compared with LCP values, may be explained by the lower incidence of exception values, with only 0.5 % of the child array values in the fly genome being 255 or greater, 0.3 % in the chicken genome, and 0.4 % in the human genome.

We then tested our algorithmic variation (Algorithm 3) that uses a DC array to retrieve genomic characters, rather than a genome-based approach (Algorithm 2) that accesses the suffix array and then the genome string. The DC-based approach gave a substantial improvement in speed, by a factor of 1.7 over the genome-based approach using bytecoding and a guide interval of 1024.

### ESA variants

Since our ultimate objective is to accelerate genomic search, we performed experimental tests on overall search speed, using various methods that were found to be efficacious in our separate LCP retrieval and child selection benchmarks. We wrote a benchmark program for search speed that, on each trial, generated 1 million random query sequences from a given genome, with lengths of 12, 24, and 36 nucleotides per sequence. Each method was then used to locate the genomic positions of these query sequences. Our methods of interest involved (1) the child and LCP arrays represented as uncompressed vectors (ESA); (2) bytecoding of both arrays without an EG array (ESA-byte); (3) bytecoding with EG arrays at a guide interval of 1024 on both the child and LCP arrays; (4) the same bytecoding format with EG arrays, plus a discriminating character array; and (5) the two bytecoding arrays with EG arrays, and the DC array integrated into a single data structure (ESA-gdi). For methods (1) through (4), we used the genome_esa class that we added to the SDSL package. These classes implement bytecoding, with or without EG arrays, for representing the LCP and child arrays. The results for ESA-gdi derive from the genome_integrated class that we added to SDSL, with guide intervals of 1024.

**Fig. 5:**
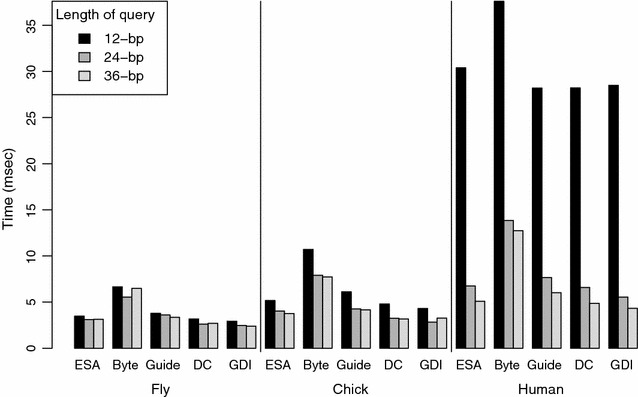
Timing results for variants of enhanced suffix arrays. The graph shows times in ms per query for the genomes tested: fly (dm5), chicken (gg4), and human (hg19). Within each genome, methods tested are: integer vector representations of the LCP and child arrays (ESA); bytecoding of both arrays (Byte); bytecoding with exception guide arrays at intervals of 1024 (Guide); addition of a discriminating character array (DC); and using an integrated data structure that combines the LCP, child, and DC arrays (GDI). For each method, timing is measured for 12-, 24-, and 36-nucleotide patterns

**Fig. 6:**
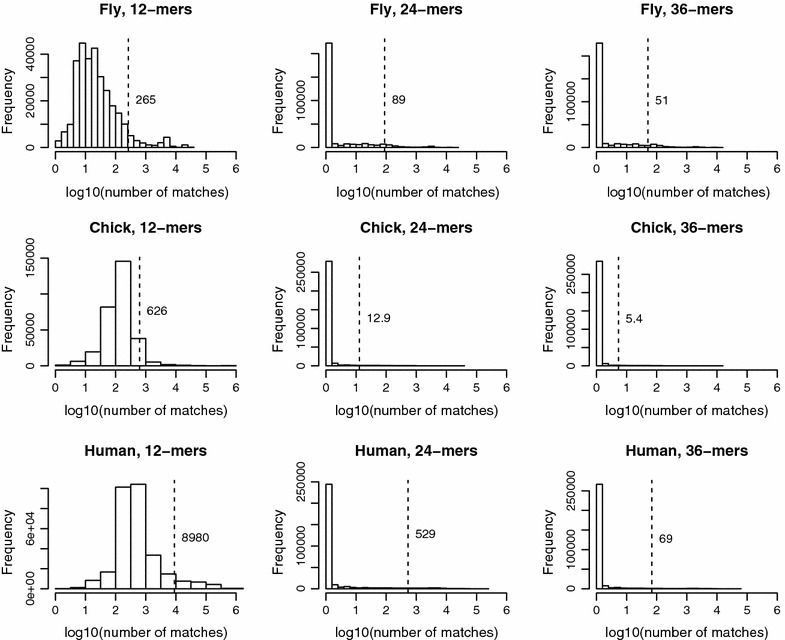
Distributions of search results. The histograms show the number of matches retrieved for various oligomer sizes in three genomes: fly (dm5), chicken (gg4), and human (hg19). Each histogram is computed over 100,000 randomly generated oligomers from the respective genome. The number of matches is shown on a logarithmic scale. The arithmetic mean of each distribution is shown by a *dashed vertical line*, and the numerical value is shown next to the line

Figure 5 shows the resulting search times. Times increase from the fly to chicken genome, and from the chicken to human genome. This finding reflects primarily the number of oligomers retrieved, with more oligomers of a given size found in larger genomes. To account for this factor, we tallied the size distributions of match results. Figure 6 illustrates these distributions on a logarithmic scale, as well as their arithmetic means, shown with a dashed vertical line. The shapes of the distributions show that 12-mers are non-specific in all species, requiring hundreds to thousands of results to be retrieved on average for each query. In particular, the large number of match results for human 12-mers accounts for the relatively long search times for that test scenario. The distributions for 24-mers and 36-mers show a high frequency of specific, or singleton, matches, but a long tail to the right, indicating that occasional 24-mers and 36-mers are non-specific and yield large numbers of match results. These non-specific oligomers generally come from duplicate or repetitive regions of the genome.  The greater overall specificity for the chicken genome, compared with the fly or human genome, may reflect its relative paucity of DNA repeats, duplications, and noncoding DNA [24]. To illustrate this difference, the chicken genome has approximately the same number of coding genes as the human genome, but is only one-third the size.

Bytecoding by itself results in slower search times compared with the uncompressed child and LCP arrays, by a factor of 1.8–2.5. However, addition of EG arrays improve the search times to be nearly those for the uncompressed ESA. To put it another way, EG arrays improve search speed by a factor of 1.5–2.0, compared with bytecoding alone.

The DC array gives additional increases in search speed, such that search becomes faster than for the uncompressed ESA by a factor of 1.0–1.2. Finally, the use of an integrated data structure, to produce ESA-gdi, gives the greatest amount of speed up, with speeds that are 1.1–1.4 times faster than those for the uncompressed ESA. Compared with the bytecoding method, the integrated data structure gives speeds that are 2.4–2.8 times faster for the fly genome, 2.2–2.7 times faster for the chicken genome, and 1.3–2.9 times faster for the human genome, over the various 12-, 24-, and 36-nucleotide pattern lengths.

### Survey of approaches

We compared our ESA methods against existing methods from the literature. The results, shown in Table 1, were obtained by creating timing experiments for backward and forward search algorithms as implemented within SDSL. For the backward search algorithms, we used the count and locate algorithms as implemented within the suffix_array_algorithm class. The protocol was the same as for our ESA benchmarking experiment, except that each experimental run was based on 100,000 randomly selected reads, instead of 1 million. Also, to obtain higher accuracy for these results, we performed 27 runs of each combination before isolating the median value.

The standard suffix array method (shown as SA in Table 1) was benchmarked using the csa_bitcompressed class implemented in SDSL, which allocates a uniform number of bits to every entry in the suffix array. The backward search algorithm uses either a successor array called Psi [10] or rank queries on a wavelet tree [11], while the forward search algorithm uses binary search through the suffix array.

Compression of the suffix array (shown as CSA-Sada and CSA-WT) was tested using the Sadakane [10] and wavelet tree [11] methods as implemented in the csa_sada and csa_wt classes, respectively. Parameters for these methods were sampling densities of 10 for the suffix array and 10 for the inverse suffix array, with sampling performed using text order rather than suffix array order. These parameters were suggested by the author of the SDSL package (personal communication), because default parameters (sampling density of 32 for the suffix array and 64 for the inverse suffix array) gave search speeds for the location task that were prohibitively slow. We also tested the wavelet tree method with an uncompressed suffix array (shown as SA-WT), by using a sampling density of 1.

Overall, these results show a tradeoff between the size and speed of a genomic representation. The smallest memory usage is provided by compressing the suffix array. The CSA-Sada method has approximately the same speed for the counting task as the uncompressed SA, whereas CSA-WT is significantly faster.

For the locating task, we find that the overall time per query correlates with the number of locations that must be enumerated. As we saw with the ESA variants and Fig.  6, the size of the match results varies significantly among the species and oligomer lengths. Therefore, in Table  1, we normalize the locating task times by the total number of match results for each condition, to obtain time per match.

Both the overall and normalized results show that the uncompressed SA is relatively slow for specific queries, such as 24-mers and 36-mers, when its speed is dominated by traversing the suffix array, as reflected in the counting task. In these cases, CSA-WT was faster than uncompressed SA. However, for non-specific queries, such as 12-mers, the compressed suffix array methods are slower than uncompressed SA, since they require additional time for decompressing large numbers of match results.

The fastest method in almost all cases of the counting and locating tasks was ESA-gdi, which uses about twice as much memory as an uncompressed SA. Of note, ESA-gdi was even faster than the uncompressed ESA, even though the compression reduces memory usage by 40 %. An exception held in the category of the counting task for 12-mers, in which the SA-WT and CSA-WT methods were slightly faster than ESA-gdi.

## Discussion

In this paper, we have explored practical issues of designing algorithms and data structures for fast genomic search. In general, we observe that more complex approaches to compression, such as permuted LCP arrays and balanced parenthesis representations of child arrays, can occupy the smallest amounts of space but require significantly more time for decoding. In practice, this additional time can limit their utility for performing tasks such as high-throughput read alignment. In contrast, simpler bitpacking and bytecoding techniques are not as efficient in space usage but much faster for retrieval time and search speed.

In this paper, we have introduced three techniques for achieving improved speed and reduced space requirements for pattern search using enhanced suffix arrays. First, for both LCP and child arrays, bytecoding when combined with an EG array provides the fastest retrieval and selection times. These findings are further supported by the observation that search with EG arrays is faster than with bytecoding itself by a factor of 1.5–2.0. Bytecoding with EG arrays is even faster than vectorized bitpacking, which was extremely effective in our companion paper on representing hash tables. One reason for this is that LCP and child arrays require direct coding of their values, rather than differential coding of differences between adjacent values. Therefore, vectorization, or SIMD, operations provide little advantage in this scenario.

Our second technique introduces a discriminating character (DC) array to speed up genomic search by reducing accesses to the suffix array and genome string. Third, integrating the LCP, child, and DC arrays into a single data structure achieves further speed up in pattern matching by improving memory access. Our integration scheme dovetails with our two previous techniques. Bytecoding of both the LCP and child arrays provides a uniform representation of small values that can be easily interleaved. Also, our use of a DC array eliminates accesses to the suffix array and genomic text, so that they do not need to be included in our integrated data structure.

Our work has been guided by our application of interest, namely, genomic search, and it is an open question whether our techniques will necessarily generalize to other domains. Genomic pattern matching is characterized by large numbers of match results per query. Even for relatively long oligomers, such as 24-mers and 36-mers, which yield only a single match result in most cases, occasional oligomers are non-specific and require many match results to be enumerated. Therefore, in this domain, it appears better to leave the suffix array itself uncompressed for fast retrieval in the locating task. Likewise, genomic pattern matching is characterized by a small alphabet size. It is not clear whether larger alphabets will benefit from a DC array if there are many child lcp-intervals for a given parent lcp-interval, which is potentially $O(|\Sigma _S|)$. In those domains, it may be faster to have a representation of the lcp-interval tree that allows the correct child to be found in $O(\log |\Sigma _S|)$ time [16, 25].

Nevertheless, our results provide guidance for the important and timely task of designing genomic search algorithms. In particular, suffix array algorithms require the most time for the locating task when many positions must be enumerated, as with the 12-mers studied in this paper. Therefore, for such queries, other representations, such as hash tables, may be more effective. In fact, our genomic alignment program gsnap [8] uses both suffix arrays and hash tables to perform genomic alignment. The techniques reported in this paper and its companion were motivated by an attempt to increase the speed of that program. Genomic search remains an important domain of interest for computational and experimental biologists. We hope that our techniques will facilitate the analysis of ever-increasing volumes of data from high-throughput sequencing.

## Additional files

10.1186/s13015-016-0068-6 Source code for methods: Source code in an archive format, using tar and bzip2, for all implementations of enhanced suffix arrays, based on a modification of the SDSL 2.0 package. Package also includes benchmarking programs used for timing experiments. This is the same package as provided in our companion paper on bitpacking for hash tables.
10.1186/s13015-016-0068-6 Source code for constructing benchmarks: Source code in an archive format, using tar and bzip2, for users to generate their own benchmarks for any genomic text in FASTA format. This is the same package as provided in our companion paper on bitpacking for hash tables.
